# A two-stage spatial allocation model for elderly healthcare facilities in large-scale affordable housing communities: a case study in Nanjing City

**DOI:** 10.1186/s12939-018-0898-6

**Published:** 2018-12-12

**Authors:** Tiantian Gu, Lingzhi Li, Dezhi Li

**Affiliations:** 1 School of Civil Engineering, Southeast University, Jiangning District, Nanjing, 211189 China; 2 Lyles School of Civil Engineering, Purdue University, West Lafayette, IN 47907 USA; 3 School of Civil Engineering, Nanjing University of Technology, Nanjing, 211816 China

**Keywords:** Affordable housing, Elderly healthcare facility, Spatial allocation, Geographic information system, Greedy algorithm

## Abstract

**Background:**

As the proportion of elderly residents living in large-scale affordable housing communities (LAHCs) increases in China, serious problems have become apparent related to the spatial allocation of elderly healthcare facilities (EHFs), e.g., insufficient provision and inaccessibility. To address these issues, this study developed a location allocation model for EHFs to ensure equitable and efficient access to healthcare services for the elderly in LAHCs.

**Methods:**

Based on discrete location theory, this paper develops a two-stage optimization model for the spatial allocation of EHFs in LAHCs. In the first stage, the candidate locations of EHFs are specified using geographic information system (GIS) techniques. In the second stage, the optimal location and size of each EHF are determined based on the greedy algorithm (GA). Finally, the proposed two-stage optimization model is tested using the Daishan LAHC in Nanjing, Eastern China.

**Results:**

The demand of the elderly for accessibility to EHFs is in line with Nanjing’s planning standards. Deep insights into spatial data are revealed by GIS techniques that enable candidate locations of EHFs to be obtained. In addition, the model helps EHF planners achieve equity and efficiency simultaneously. Two optimal locations for EHFs in the Daishan LAHC are identified, which in turn verifies the validity of the model.

**Conclusions:**

As a strategy for allocating EHFs, this two-stage model improves the equity and efficiency of access to healthcare services for the elderly by optimizing the potential sites for EHFs. It can also be used to assist policymakers in providing adequate healthcare services for the low-income elderly. Furthermore, the model can be extended to the allocation of other public-service facilities in different countries or regions.

## Background

An elderly healthcare facility (EHF) is a place where healthcare services are provided for the elderly (aged 60 and over), and such facilities include community healthcare centers and community healthcare clinics [1]. The former mainly provide family-oriented healthcare services for the elderly, such as primary and preventive healthcare as well as links to welfare (e.g., providing life consulting services for the elderly, providing mental care for the elderly, and regularly organizing sports activities for the elderly), while the latter are branches of community healthcare centers that serve as supplements to them [2–4]. These facilities not only provide a wide range of benefits for the elderly but also provide services for individuals who are unable to care for themselves because of mental or physical infirmity. In reality, most of the EHF users in LAHCs are the elderly. The elderly generally tend to have a higher prevalence of chronic diseases, mental illnesses and other comorbidities [5]. Therefore, primary and preventive healthcare provided by EHFs are essential to maintaining health for the elderly. In addition, a long travel distance to obtain healthcare services affects the accessibility of EHFs. Hence, allocating EHFs is an important part of the elderly healthcare system [6, 7]. Demographic changes have been one of the major factors in the expansion of EHFs in China [1, 8]. During the period from 2015 to 2050, the proportion of the world’s population over 60 years of age will almost double from 12 to 22%, and China is aging faster than almost any country in recent history. By 2050, the population aged 60 years or over in China is expected to exceed 35% [9]. To address the country’s severe aging problem, “The 13th Five-Year National Plan (2016-2020) for Developing the Elderly Care System” was issued in 2017. It requires that home-based care for the elderly population be prioritized, and correspondingly, more EHFs should be provided in communities [10]. However, recent studies have illustrated many misallocation problems regarding EHF planning and resource allocation, such as insufficient provision and poor accessibility [11, 12]. For example, the majority of the villages of Yanqing District in Beijing have relatively poor accessibility to healthcare services due to the uneven distribution of EHFs [13] .

These problems are even worse in large-scale affordable housing communities (LAHCs) in China because most LAHCs are constructed far from traditional commercial centers, and the facilities provided in such communities cannot satisfy the needs of the elderly [14, 15]. It is well known that during the period from 2011 to 2015, affordable housing projects were vigorously advocated, with the aim of resolving the growing housing problems for low-income people in China [16, 17]. Considering economic motivations, local governments constructed many LAHCs with construction areas of more than 1 million square meters in remote locations during this period [18]. The critical issue is that the proportion of the elderly in these communities is higher than that in other residential communities [19]. For example, by the end of 2016, the proportion of the elderly in the Daishan community (one of the LAHCs in Nanjing) had reached 30%, which far exceeded the overall proportion of the elderly in Nanjing (19.98%) [20]. This phenomenon has mainly occurred because many landless elderly people moved to LAHCs after their land was expropriated [21]. Thus, access to healthcare services (i.e., spatial accessibility to EHFs) became a public issue, and the demand has increased in LAHCs. Therefore, the allocation of healthcare resources for the elderly in an equitable and effective way has become urgent and critical [18, 22]. However, the existing EHFs were allocated based on outdated planning standards that did not consider the concentration of the elderly in LAHCs [23]. This approach has resulted in the unavailability, inaccessibility, and insufficient provision of healthcare services for the elderly in LAHCs [19, 23]. Therefore, a systematic model for the allocation of EHFs in LAHCs is urgently needed.

Previous studies on the allocation of healthcare facilities are mainly based on discrete location theory in which the objectives of constraints to allocation are critical elements [24–26]. In general, the attractiveness of healthcare facilities, environmental goals based on land use, and improving the equity and efficiency of access to healthcare facilities have been considered the primary objectives in resolving location-allocation problems [6, 27–30]. Additionally, various constraints that influence the allocation of facilities have been considered, such as the catchment areas of existing healthcare facilities, land use constraints and the elderly’s demand for healthcare services [31, 32]. To enrich this theory, the maximal covering location planning (MCLP) model and the P-median (PM) model have been frequently applied in empirical studies of healthcare allocation problems [24, 33, 34]. The MCLP model attempts to maximize the population to be served given a limited number of fixed facilities [35–37]. However, the model may lead to inefficiencies because travel distance is not considered [24]. In contrast, the PM model was developed to determine the optimal spatial distribution to minimize citizens’ average travel distance or travel time [38, 39]. Worst-case situations cannot be incorporated into the model, and equitable access cannot be guaranteed [6]. In addition to these models, other models have been recently proposed, such as the p-dispersion model [33]. Although location allocation models are NP-hard (nondeterministic polynomial hard), they can be solved by a variety of algorithms, both optimally and heuristically, such as the genetic algorithm [40], the hybrid artificial bee colony algorithm [41], and the greedy algorithm. In cases of allocation models with multiple objectives, the greedy algorithm has been found to yield better results compared to other algorithms [35]. In addition, with the development of computing technology, geographic information system (GIS) techniques have been widely employed to develop optimal solutions to allocation problems [2]. Many scholars have used GIS techniques as a way to define healthcare catchment areas, obtain optimal locations and analyze accessibility to healthcare facilities [31, 42, 43]. Such techniques can provide a more realistic measure of spatial information [44–46].

However, many challenges have been identified in previous studies. First, limited attention has been paid to the actual demands of end users when allocating healthcare facilities. Second, few studies have simultaneously focused on ensuring equitable and efficient access to EHFs for the elderly. Third, there are few alternative solution methods that have been proposed to solve models with multiple objectives, and the greedy algorithm has not been fully explored. In addition, the application of GIS is still at a low level in this research field. Related spatial information (e.g., the elderly population, land use constraints and existing facilities) has not been fully incorporated into the prevailing facility allocation models, which results in inaccurate or vague results [47, 48]. Nevertheless, location modeling of EHFs in LAHCs is an exciting area of study for researchers and practitioners.

To fill these gaps, this paper aims to develop a two-stage spatial allocation model for EHFs in LAHCs by combining the objectives of equity and efficiency. The specific research questions are as follows: (1) What is the elderly’s actual demand for spatial accessibility of EHFs in LAHCs? Is this demand consistent with the demand for spatial accessibility in related planning standards? (2) How can candidate locations of EHFs be obtained in an LAHC? Can this process be presented in a visual way? (3) How can the optimal locations of the EHFs be determined? Can this model achieve the bi-objectives of simultaneously ensuring equitable and efficient access to healthcare services for the elderly?

The present study has the following structure. In Section 2, the two-stage model is proposed, and the study area is described. In Section 3, the results are presented by applying the proposed model to the case of Nanjing, China. In Section 4, the designed solution procedure is discussed. In Section 5, the strengths of the study are presented, and potential topics for future studies are proposed.

## Methods

**Fig. 1:**
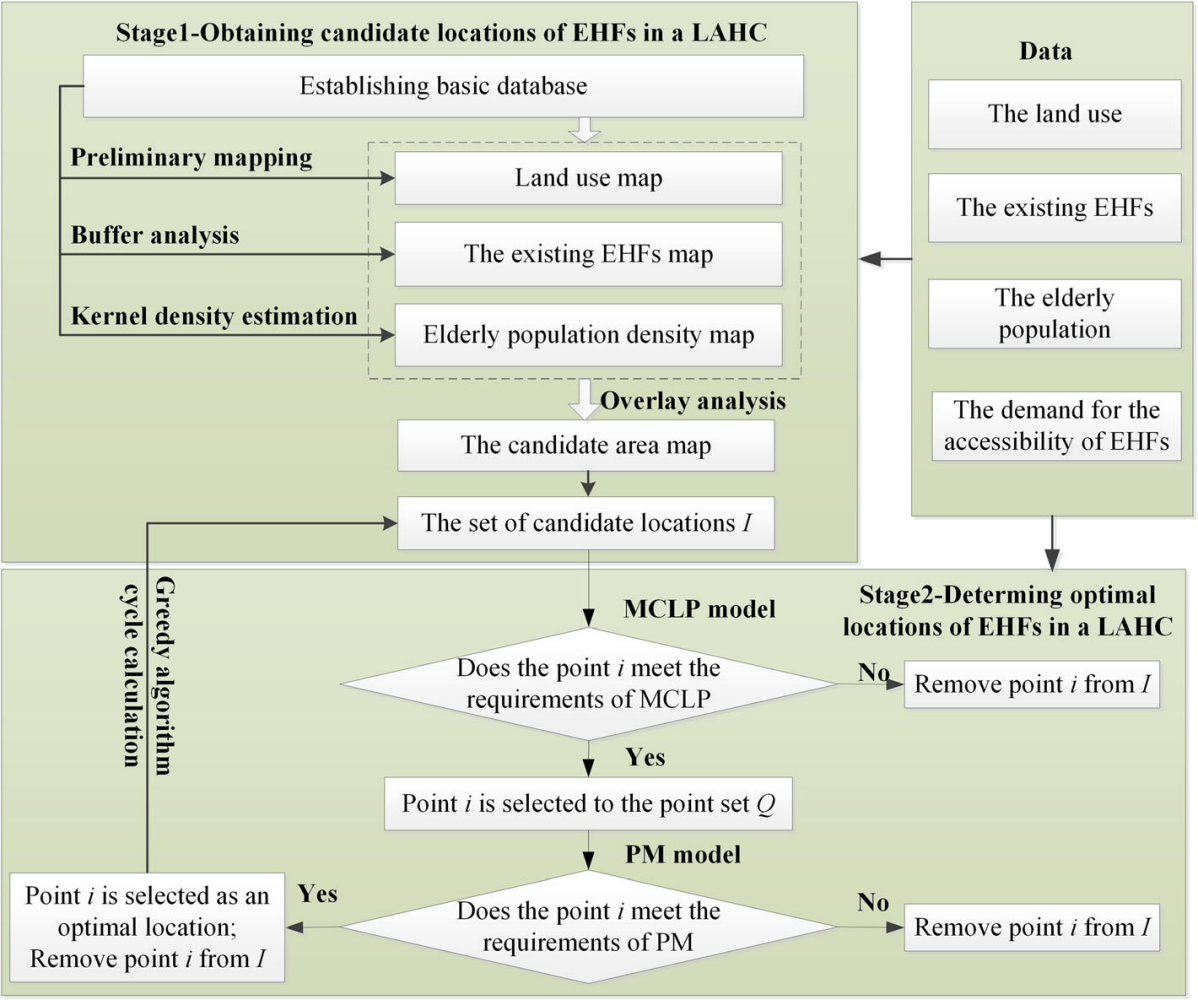
General flow diagram of computational framework

Based on current location allocation problems of EHFs in LAHCs and the reviewed literature, a two-stage spatial allocation model was formed with the bi-objectives of covering as many old people as possible within the catchment areas of EHFs and achieving the shortest distance from the demand points to the facility. This analysis includes four types of data: (a) land use, (b) existing EHFs, (c) the elderly population, and (d) the elderly’s demand for accessibility to EHFs. The computational framework of the model is shown in Fig. 1, in which a flow diagram illustrates the individual stages. Detailed information about this model will be discussed in the following sections.

### Stage 1 - obtaining candidate locations of EHFs

In the first stage, the location scope of an LAHC is narrowed using four GIS techniques (preliminary mapping, buffer analysis, kernel density and overlay analysis). This process involves four types of data. The elderly’s demand for accessibility to EHFs is determined through questionnaires. The key question is, “How far are you willing to walk to an EHF in your community?” The corresponding options are “A) within 5 minutes, B) 5-10 minutes, C) 10-15 minutes, D) 15-20 minutes, and E) over 20 minutes”. Spatial information data are obtained from the government or the developers of LAHCs. ArcGIS software is used for these functions.

First, we established a basic database of LAHCs. Using drawing tools, a land use map, existing EHF map and a map of the elderly population were developed to obtain spatial information about the LAHCs. Then, the collected attributes of the point, polyline and polygon were input into the model. The point features usually included the elderly population, the community ID and the locations of the EHFs. The polyline features included the length of the roads. The polygon features included the elderly population, the number of residential units, the community ID and the land area. Among these data, the elderly population is particularly important. The formula used to calculate the elderly population at the center of each building is shown in formula (1):

1 $$ {EP}_i=\left(\frac{a_i}{A}\right)\ast P\ast N\ast R $$

where.

*EP*_*i*_: the elderly population at the center of building *i*;

*a*_*i*_: the floor area of building *i*;

*A*: the total floor area of the buildings in the community;

*P*: the number of households in the community;

*N*: the average number of people per household;

*R*: the proportion of the elderly population;

*i*: the number of buildings.

Second, we defined the catchment areas of the existing EHFs through buffer analysis and by obtaining a map of the existing EHFs. Because buffer analysis can generate the features of an area at a specified distance (or at several specified distances) around the input features [49], it is widely used to define protected zones around features or to show the scope of influence of certain features. By placing an existing EHF at the center of a circle, its catchment area can be calculated within a certain radius. In this paper, *M* and *m* are used as the service radii of community healthcare centers and community healthcare clinics, respectively. These facilities should simultaneously meet the elderly’s demand for spatial accessibility to EHFs and the planning standards for EHFs.

Third, we identified the spatial distribution of elderly people through kernel density estimation and obtained an elderly population density map. Kernel density can be used to estimate the data frequency by summing a set of Gaussian distributions, and it does not take into account analytical uncertainty [50]. Let *x*_*i*_(*i* = 1, 2, …, *n*) be an independent sequence drawn from an arbitrary probability distribution; the form of the kernel density estimation can then be given in formula (2) [51]:

2 $$ \widehat{f}(x)=\frac{1}{N^{\prime }h}\sum \limits_{i=1}^NG\left(\frac{x-{x}_i}{h}\right) $$

where $\widehat{f}(x)$ is the probability density function;

*x* is the elderly population at the center of the building;

*N*^′^is the sample number;

*G*(*x*)is the kernel function, and the calculation of *G*(*x*) is given as follows:

2.1 $$ G(x)=\frac{1}{\sqrt{2}}\exp \left(-\frac{1}{2}{x}^2\right) $$

*h* = the bandwidth, which is usually defined as the minimum of the mean integrated square error (MISE) [44]. The calculation of MISE is as follows:

2.2 $$ MISE=\underset{a}{\overset{b}{\int }}E{\left(\widehat{f}(x)-f(x)\right)}^2 dx $$

In formula (2), $\widehat{f}(x)$ is the estimated value of the sample points, *f*(*x*) is the true value of the same sample points, and *E*(*x*) denotes the expected value with respect to that sample.

Fourth, overlay analysis was conducted to determine the candidate locations of the EHFs. The overlay analysis can be implemented in three steps. a) First, the raster data must be reclassified, including the land use, the existing EHFs, and the elderly population density (Table 1). Because the land that is being used for other functions cannot be considered to construct a new EHF, these areas can be set to the value 0, and unused areas can be set to the value 1. Similarly, the candidate locations cannot be within the catchment areas of existing EHFs. The value 1 indicates locations that are more than *M(m)* meters away from existing EHFs; otherwise, the value is 0. Finally, this study divided the elderly population density into ten categories, represented by values 1–10. The value 1 represents the lowest elderly population density, and 10 represents the highest elderly population density. b) The second step involves overlaying the existing land use raster map, the existing EHF raster map and the elderly population density raster map to obtain a candidate area map using Map Algebra, which is a computational tool in ArcGIS Spatial Analysis. The greater the value obtained from the calculated results, the higher the suitability of the area is as a candidate. c) The third step involves determining the set of candidate locations for the EHFs based on grid analysis. A grid spacing of 25 m is used on the candidate area map, and then the point features are rasterized and reclassified. We set the value at 0 if there is no grid point, and 1 if there is a grid point. Then, we overlay the grid map with the candidate area map. Final calculated values greater than 0 indicate that the candidate points meet the above requirements. Thus, the set of candidate locations *I* is formed.

**Table 1:** Reclassification rules

Layer	Judgment	Condition	Value
Existing land use	Whether the land is currently used	Unused area	1
		Used area	0
Existing elderly healthcare center	Whether the location is more than *M*meters away from an existing center	Yes/Areas where services are not available	1
		No/Areas where services are available	0
Existing elderly healthcare clinic	Whether the location is more than *m*meters away from an existing clinic	Yes/Areas where services are not available	1
		No/Areas where services are available	0
Elderly population density	Whether the elderly population density is high	Yes/The greater the density, the higher the value is.	> = 5
		No/ The lower the density, the lower the value is.	< 5

### Stage 2 - determining the optimal locations of EHFs

The GA (greedy algorithm) is often used to solve a large number of location problems because it does what is best at each step of the algorithm without looking ahead to see how the current decisions will impact later decisions and alternatives [52]. In a wide range of practical applications, it has been shown that the local optimal solution obtained by GA can be regarded as an approximate solution to a bi-objective optimization problem [53]. Because this study examines a bi-objective optimization problem, GA is used to evaluate how much demand each candidate site covers (MCLP) and to select the optimal locations to minimize average travel distances (PM) [33]. Then, the floor area of potential EHFs is determined based on the planning standards for elderly facilities and the number of elderly people covered. The steps for the improved GA in this study are as follows.

Step 1: Calculate the maximum coverage points in the candidate point set *I* to form the point set *Q*_*i*_ based on the MCLP model, as shown in formula (3):

3 $$ \mathit{\operatorname{Max}}={\sum}_{k=1}^n{\sum}_{i=1}^m{p}_i\ast {Popu}_k $$

3a $$ S.t.{p}_i\ast {Dist}_{ik}\le M(m)\kern1.5em \forall k\in K $$

3b $$ \sum \limits_{i\in I}{x}_{ki}=1,\forall k\in K $$

$$ {p}_i\in \left\{0,1\right\}\forall i\in I $$

where *K* is the set of demand points in candidate areas;

*I* is the set of candidate locations;

*Popu*_*k*_ is the number of elderly people at demand point *k*;

*Dist*_*ik*_ is the distance from demand point *k* to facility point *i*;

*M*(*m*) is the service radius of the EHF;

$$ {p}_i=\left\{{}_{0\ \mathrm{Not}\ \mathrm{selected}\ \mathrm{to}\ \mathrm{the}\ \mathrm{point}\ \mathrm{set}\ {Q}_i}^{1\ \mathrm{Selected}\ \mathrm{to}\ \mathrm{the}\ \mathrm{point}\ \mathrm{set}\ {Q}_i}\right. $$

Inequality constraint (3a) indicates that the distance from a demand point to the candidate facility must be less than the service radius of the candidate facility. If the decision variable *p*_*i*_ equals 1, *i* is selected to the point set *Q*_*i*_. Otherwise, it will not be selected to the point set *Q*_*i*_.

Step 2: Calculate the points with the shortest distance in *Q*_*i*_ based on the P-median model. These points are considered more accurate candidate points.

4 $$ \mathit{\operatorname{Min}}={\sum}_{i=1}^t{Q}_i\ast {Popu}_k\ast {Dist}_{ik} $$

4a $$ S.t.{\sum}_{i=1}^t{Q}_i=1\kern1em \forall i\in {Q}_i $$

$$ {Q}_i\in \left\{0,1\right\}\forall i\in {Q}_i $$

where *k* is the set of demand points in candidate areas;

*Popu*_*k*_ is the number of elderly people at demand point *k*;

*Dist*_*ik*_ is the distance from demand point *k* to facility point *i*;

$$ {q}_i=\left\{{}_{0\ \mathrm{Not}\ \mathrm{selected}\ \mathrm{as}\ \mathrm{the}\ \mathrm{optimal}\ \mathrm{location}}^{1\ \mathrm{Selected}\ \mathrm{as}\ \mathrm{the}\ \mathrm{optimal}\ \mathrm{location}}\right. $$

The objective function (4) indicates that the demand point population has the smallest weighted distance to the candidate facilities. Equality constraint (4a) indicates that only one optimal candidate point can be found in each iteration of the loop. If the decision variable *Q*_*i*_ equals 1, point *i* is selected as the optimal location to construct the EHFs. Otherwise, it will not be selected as the optimal location.

Step 3: If point *i* meets the requirements of covering as many elderly people as possible and minimizing the average travel distance, then it can be removed from the set of candidate locations *I*. Then, let *i* = *i* + 1, and go to Step 1 to conduct the cycle calculation again until all point *i* that meet the requirements are obtained.

### Study area

**Fig. 2:**
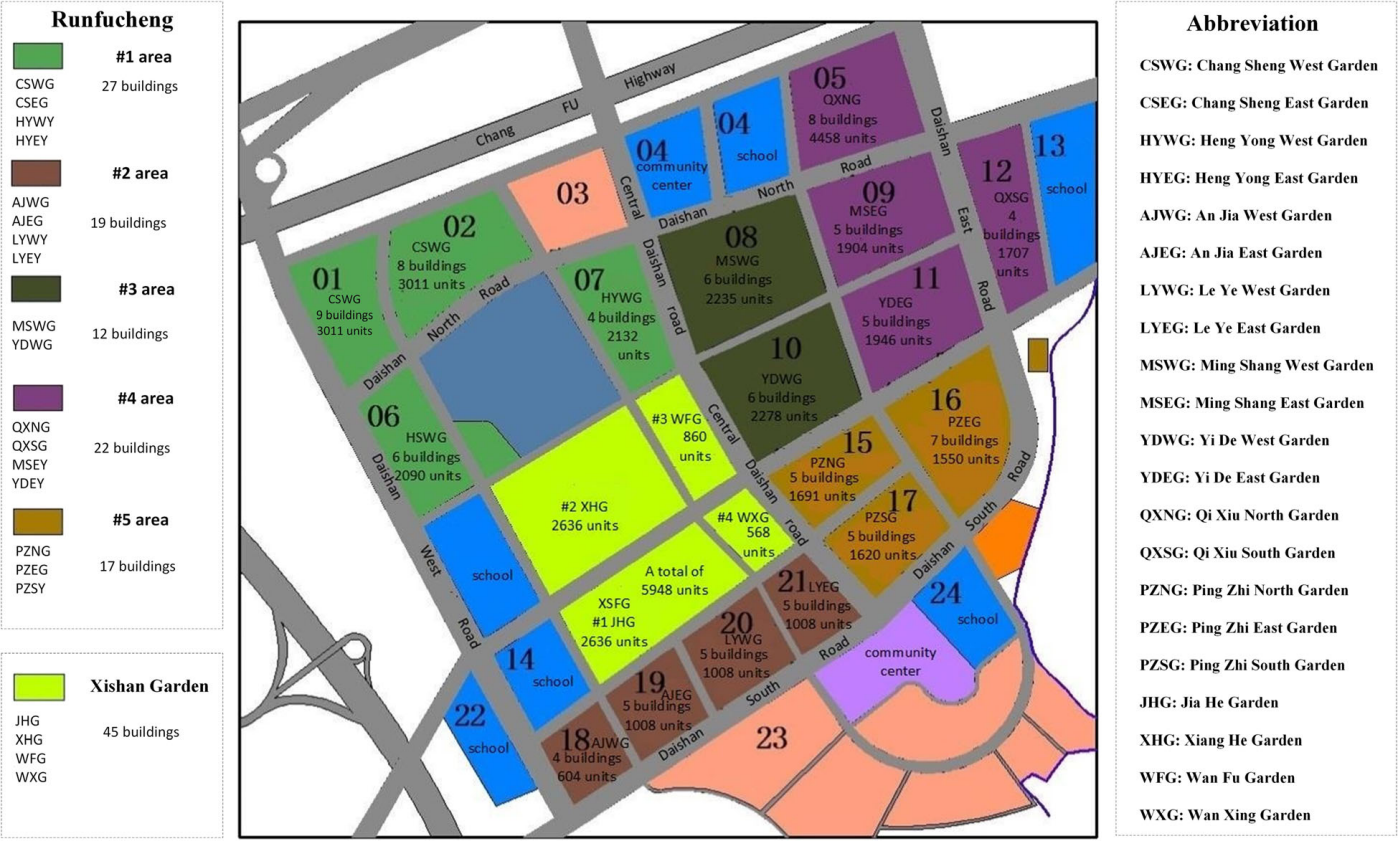
Distribution of buildings in Daishan LAHC

Nanjing, a city in eastern China, is located in Jiangsu Province. This research selected the Daishan LAHC in Nanjing City as the study area for two reasons. First, by the end of 2016, the proportion of the elderly in the Daishan LAHC was more than 30%, which is much higher than the overall proportion of the elderly in Nanjing (19.98%). Second, urgent demand for EHFs has been created due to insufficient EHFs and public transportation services in remote areas. Detailed information about this community was obtained from the Greentown Real Estate Company, which is one of the community’s developers (Fig. 2). The land in the Daishan affordable housing community is divided into 32 plots, including 10 commercial housing plots and 22 affordable housing plots. The newly built Runfucheng community and the old Xishan Garden are part of the Daishan LAHC. The Runfucheng community can be divided into five types of areas. There are 27 buildings in area #1, 19 buildings in area #2, 12 buildings in area #3, 22 buildings in area #4, and 17 buildings in area #5. A total of 45 buildings are located in Xishan Garden. In addition, one community healthcare center and four community healthcare clinics have been allocated to the Daishan LAHC. The community healthcare center is located at No. 1 Shengjiagang West Street (floor area: 4800 m^2^). The four community healthcare service clinics are the Daishan community healthcare clinic (floor area: 312 m^2^), which is located at No. 6 Shengjiagang Street, the Yongsheng community healthcare clinic (floor area: 740 m^2^), which is located at No. 6 Daishan North Road, the Pingzhi community healthcare clinic (floor area: 658 m^2^), which is located at No. 5 Heng Village Street, and the Xishan Garden community healthcare clinic (floor area: 820 m^2^). The elderly population at the center of building *i* (*i* = 1, 2, 3 … 45) can be calculated based on formula (1) using the data above (*R* = 30%, the proportion of the elderly population in this community is approximately 30%). All elderly people over 60 years old were selected as our research subjects. Random sampling was used to ensure that the number of observations from each community was proportional.

### Data collection

To guarantee high response rates and reliable results (i.e., to ensure that all respondents were elderly), face-to-face interviews were used to collect the data. We conducted simple interviews with the elderly while sending out the questionnaires. First, basic information on the elderly was collected, such as gender, age, health condition, and income. Then, they were asked how far they were willing to walk to an EHF in their community as well as the reason for this choice. In 2017, the total population living in the Daishan LAHC of Nanjing was approximately 100,000, and the proportion of the elderly in the Daishan LAHC was approximately 30%. Hence, the statistical theoretical sample size in the Daishan LAHC was approximately 380 with a 95% confidence level and a 5% significance level. Because there may be multiple deviations in the survey, 440 questionnaires were distributed. After the face-to-face interviews, 422 valid questionnaires were returned. The rate of effective recovery of the questionnaires was 95.91%.

## Results

According to the “Nanjing Public Facilities Planning Standards,” each community healthcare center should be allocated in an area with 30,000–100,000 residents, and its building should be 4000~5000 m^2^ (including 30–50 nursing beds). Accordingly, each community healthcare clinic should be allocated in an area with 10,000 residents. In the Daishan LAHC, the existing community healthcare center meets this planning standard because it is located in a community with approximately 100,000 residents, and its floor area is 4800 m^2^. Therefore, only community healthcare clinics are selected as the research subjects, and the allocation process is analyzed below.

### The demand for accessibility to the EHFs in the Daishan LAHC

Table 2 presents the individual characteristics of the respondents. According to the demographic and socioeconomic information, the respondents were predominately female, with average health conditions and low income. In terms of the demand for spatial accessibility, nearly half of the respondents (48.82%) were willing to walk 5 min to the community healthcare clinic. A total of 28.91% of the respondents hoped that they could arrive at the community healthcare clinic in 5–10 min, while 4.27% accepted a 15- to 20-min walk to the clinic. No one was willing to walk more than 20 min to the clinic. In the face-to-face interviews, most of the elderly people stated that they tended to have mobility problems, and longer distances could prevent them from reaching elderly facilities. One of the interviewees, a 72-year-old woman, said,

**Table 2:** Characteristics of the respondents

Characteristic		N	Freq %
Gender	Male	160	37.90%
	Female	262	62.10%
Age	60–64	54	12.80%
	65–69	64	15.20%
	70–74	102	24.20%
	75–79	88	20.90%
	> = 80	114	27.00%
Health condition	Good	122	28.90%
	All right	206	48.80%
	Bad	94	22.30%
Income	< 1000	154	36.49%
	1000–1500	64	15.17%
	1500–2000	40	9.48%
	2000–2500	40	9.48%
	> 2500	124	29.38%
Demand for accessibility to the community healthcare clinic	< 5 mins	206	48.82%
	5–10 min	122	28.91%
	10–15 min	76	18.01%
	15–20 min	18	4.27%
	> 20 mins	0	0.00%

*“I live with my husband now. Last year, I was taken to the hospital because of a leg injury. Since then, I have been unable to walk for a long time. So I hope that the EHFs could be as close as possible to me.”*

Most of the respondents would accept a 5-min walk to community healthcare clinics; that is, they were willing to walk 250–300 m to community healthcare clinics (generally, the walking speed of the elderly is 50 m~ 60 m/min). In addition, according to the “Nanjing Public Facilities Planning Standards,” the radius for community healthcare centers and community healthcare clinics should be 500–600 m and 250–300 m, respectively. Therefore, in this paper, it is appropriate to set 600 m and 300 m as the service radii of the community healthcare centers and community healthcare clinics (*M*=600, *m*=300).

### Stage 1 - obtaining candidate locations of EHFs in the Daishan LAHC

**Fig. 3:**
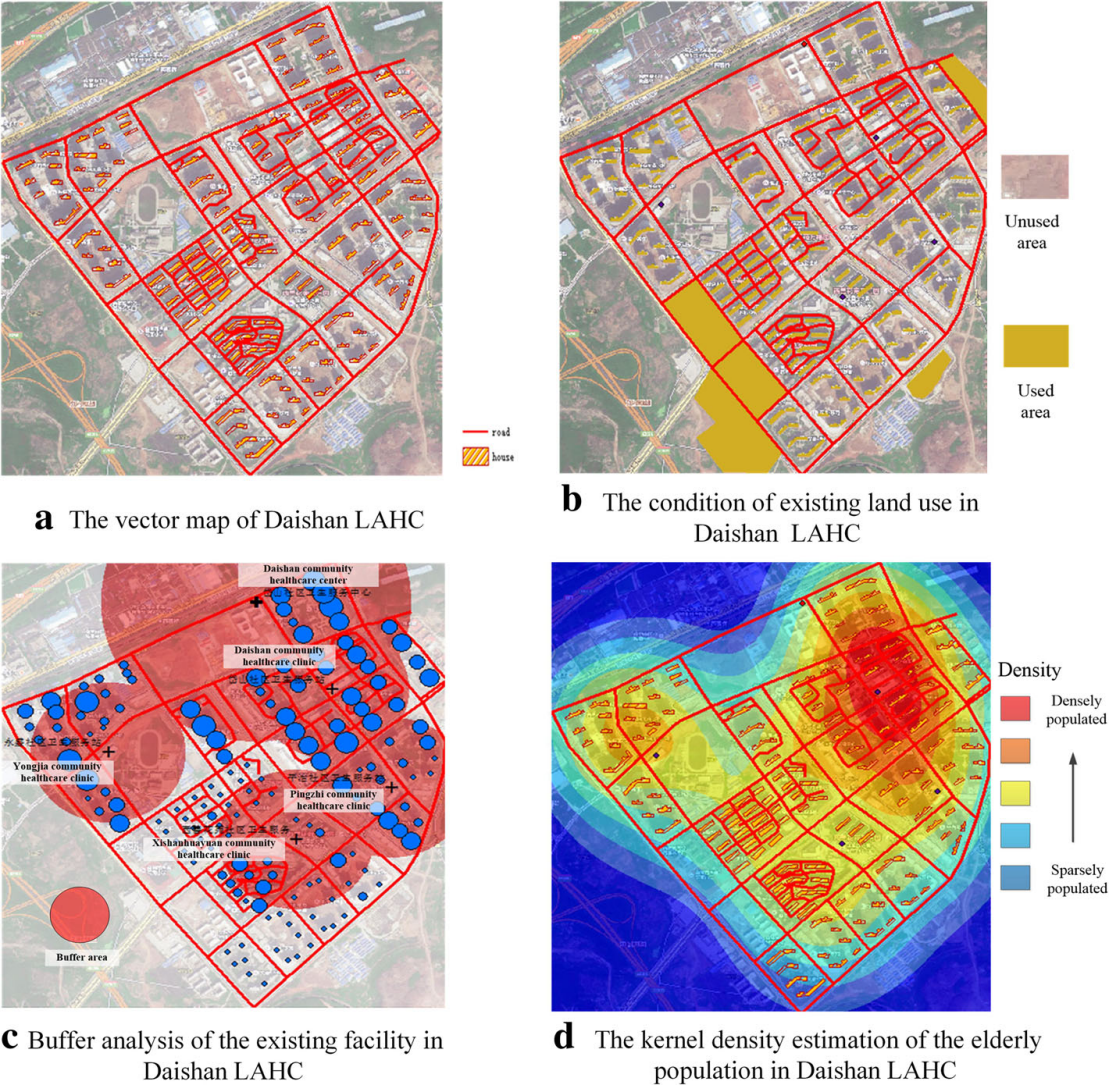
Preliminary mapping (**a** and **b**), buffer analysis (**c**) and kernel density estimation (**d**) of Daishan LAHC

**Fig. 4:**
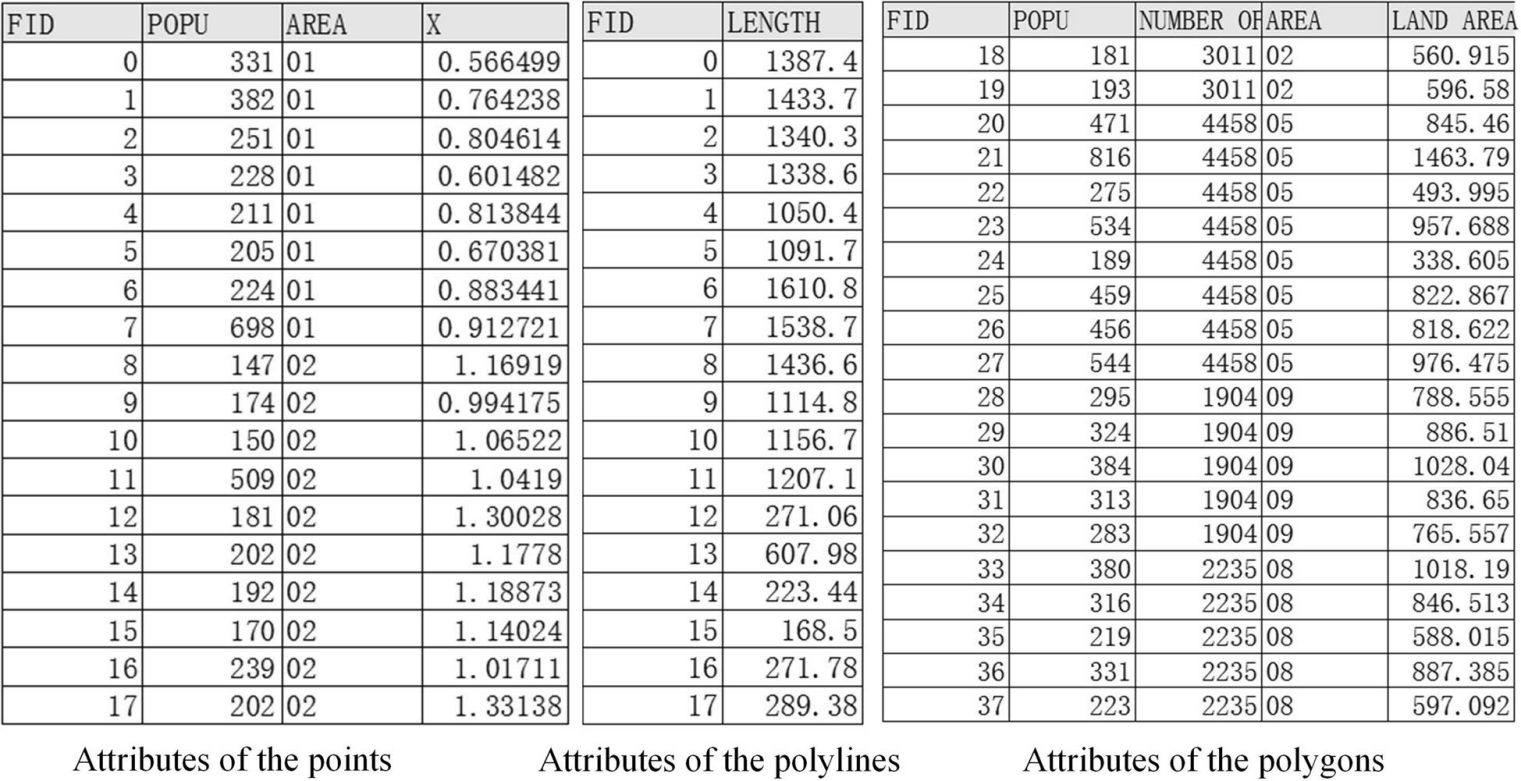
Partial data about the points, polylines and polygons

Unlike other communities, the Daishan affordable housing community does not have a database with information about its population and the existing buildings. Therefore, the first step was to obtain a vector map and construct a basic database for this area. Based on the data obtained above, the vector map was drawn, as shown in Fig. 3a. Then, all attributes of the point, polyline and polygon were input into the vector map, and the results are shown in Fig. 4.

According to the planning map and satellite map of the Daishan LAHC, some of the land areas are used for other purposes. For example, some areas have houses and schools. These plots that are already used for other purposes are generally not considered for future plans, so the condition of the existing land needs to be analyzed. In Fig. 3b, the yellow area represents used land, and the brown area indicates unused land. Therefore, the new community healthcare clinics should be built in a brown area.

Next, we defined the service radius of the one community healthcare center and the four community healthcare clinics in the Daishan LAHC using buffer analysis. Based on the results regarding the demand for spatial accessibility of the EHFs in the Daishan LAHC, the suggested service radius of community healthcare clinics and community healthcare centers should be 300 m (*m*=300) and 600 m (*M*=600), respectively. The buffer zone of these facilities is shown in Fig. 3c.

Based on the calculated data on the elderly population at the center of building *i* (*i*=1,2, 3 …,45), the kernel density of the elderly population can be estimated, as shown in Fig. 3d. The bluish area represents the lowest elderly population density, and the reddish area indicates the highest elderly population density. To serve more elderly people, the candidate locations of community healthcare clinics should be in more densely populated areas (the red areas).

**Fig. 5:**
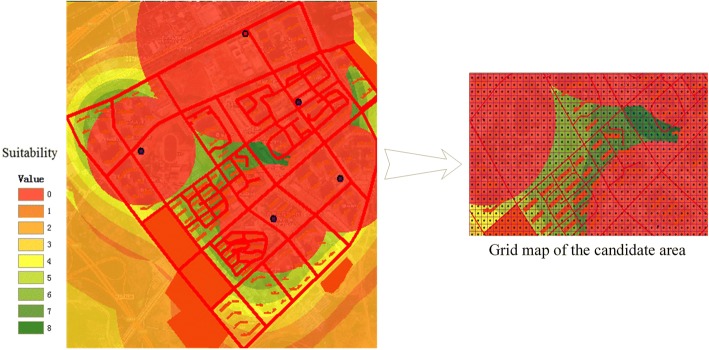
The candidate areas for the community healthcare clinics

Next, we reclassified the raster data on the existing land use, the existing elderly healthcare service area, and the elderly population. A new map that shows the suitability of candidate areas for EHFs can be obtained through overlay analysis (Fig. 5). Because value 4 is used as the threshold for the suitability index, the green area is more suitable as a candidate area. Then, a candidate area map was processed with a grid spacing of 25 m to obtain the set of candidate locations *I*.

### Stage 2 - determining the optimal locations of EHFs in the Daishan LAHC

**Fig. 6:**
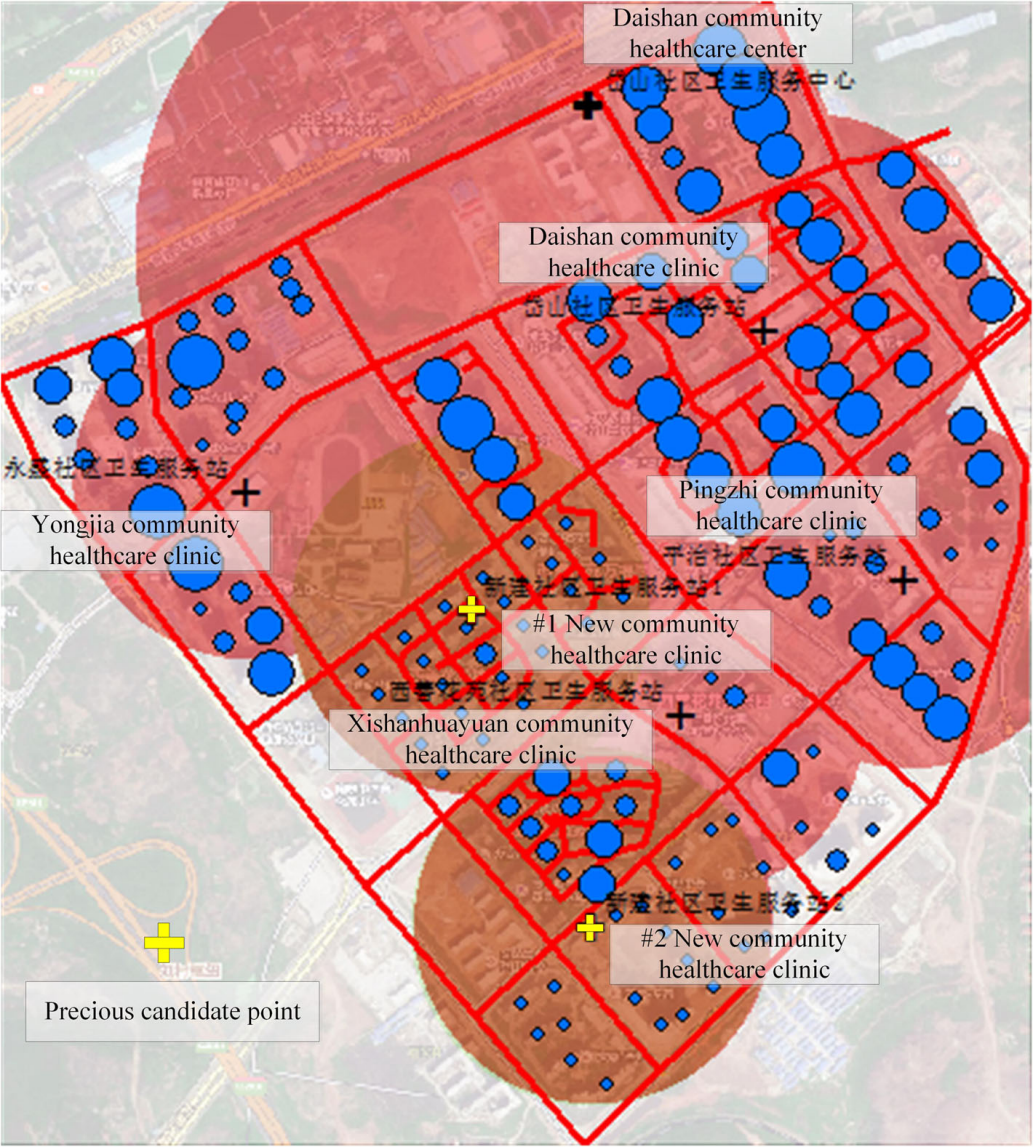
Optimal candidate locations for the community healthcare clinics

Two candidate points were obtained by conducting cycle calculations in stage 2. The first point suggested for a new community healthcare clinic was located in Xiang He Garden, and the second one was located in An Jia East Garden. The catchment areas of the new clinics and the existing EHFs are shown in Fig. 6. Approximately 12,430 people and 14,390 people are covered by the first (#1) and the second (#2) new community healthcare clinics, respectively, at a service radius of 300 m. According to the “Nanjing Public Facilities Planning Standards,” a community healthcare clinic can be allocated when the population of the area reaches 10,000, and the floor area of each clinic should be 15–30 m^2^ per thousand people. By selecting 30 m^2^ per thousand people as the index to construct new community healthcare clinics, the floor area of each community healthcare clinic can be calculated (rounded up) as follows:

The floor area of community healthcare clinic #1 = 12,430 × 30/1000 = 373 m^2^.

The floor area of community healthcare clinic #2 = 14,390 × 30/1000 = 432 m^2^.

## Discussion

In this study, we propose a two-stage optimization model for the spatial allocation of EHFs in LAHCs. Unlike traditional methods that allocate elderly facilities based only on a single objective [39], this paper defines a new model with two objectives to improve the equity and efficiency of the allocation of EHFs in LAHCs. We can be confident of a number of important findings.

First, the planning standards for the accessibility of EHFs in Nanjing are in line with the actual needs and are operable. In Nanjing, the related planning standard suggests that the service radius of EHFs should be 250–300 m for community healthcare clinics and 500–600 m for community healthcare centers. According to the interviews, most respondents accept a walking distance of 300 m (600 m) to community healthcare clinics (centers). The two indexes are thus in accordance with the “Nanjing Public Facilities Planning Standards.” In reality, there are often irrational planning standards that do not meet the demand of the elderly. Therefore, this study highlights the importance of the elderly’s demand for spatial accessibility to EHFs. If the spatial accessibility to EHFs in the planning standard are different from the actual needs of the elderly, the elderly’s demand should be met. This model helps to satisfy the demand of the elderly for healthcare services compared to the previous situation.

Second, as simple but visually powerful tools, GIS techniques have been used to obtain candidate locations of EHFs in an LAHC. In the first stage, the primary data and secondary data are input as the attributes of the point, polyline and polygon. This makes it possible to process as much spatial data as possible through GIS techniques. Figure 3(c) shows that approximately 1/4 of the area is not covered by the existing EHFs and that the catchment areas of the existing EHFs cannot serve all the elderly in the Daishan LAHC, which makes it necessary to build new EHFs in this community. As shown in Fig. 3(d), the kernel density analysis indicates that few people live on the border of this community, which is the reason why we did not consider the impact of EHFs in the neighboring community. Based on the maps with spatial attributes, the candidate area map was generated by overlay analysis. As shown in Fig. 5, the green area was chosen as the candidate area. No matter how large the research area is, it can be narrowed, and candidate areas can be obtained through the first stage. The results obtained from a visual analytic perspective can allow decisionmakers to optimally allocate EHFs according to the situation.

Third, this two-stage model makes it possible to achieve the bi-objectives of simultaneously ensuring equitable and efficient access to healthcare services for the elderly. As a consequence, the model enables the optimal locations of EHFs to be obtained by conducting cycle calculations. On the one hand, equitable access can be ensured by achieving the objective of covering as many elderly people as possible within a certain radius. On the other hand, the efficiency of the location allocation can be improved by obtaining the shortest distance from the demand point to the facility. The implementation of GA accelerated the resolution process and helped us to obtain the optimal candidate points for the EHFs in an LAHC. After obtaining the locations of the new community healthcare clinics, buffer analysis was again conducted. Figure 6 shows that the remaining demand points are completely covered by the new community healthcare clinics, which demonstrates the validity of the model. Thus, the results represent a useful asset for decision support in improving EHF planning and evidence-based policy development.

Fourth, considering the growth in the elderly population in LAHCs, the present study helps to identify areas for the expansion of existing facilities. In general, the population factor is known to have a substantial influence in the determination of EHF allocation [39]. Monitoring the growth of the elderly population is important when estimating the actual demand for EHFs in LAHCs. This study supports the view that with the increase in the housing occupancy rate, more and more elderly people will live in this community, and the utilization of EHFs will increase accordingly. Therefore, we assume that the occupancy rate of each building is 100% when calculating the elderly population at the center of each building. In this way, the existing and new EHFs can meet the need for the majority of the elderly in the near future.

However, there are a number of limitations in this type of analysis that must be considered when interpreting the results. First, to simplify the analysis, walking is considered to be the most common method of travel for the elderly in the community. Although the elderly have different choices for travelling to EHFs in reality, studies have yet to develop a method to incorporate the diverse behaviors of the elderly into an allocation model. Second, this paper takes spatial and demographic factors into consideration when allocating the EHFs in an LAHC. Little attention has been paid to other factors, such as the demand of the elderly for different types of healthcare services, the quality of services provided and the choices of family members. In addition, whether a candidate location will be accepted is determined by economic factors and environment policy factors. It is very challenging to examine all of these factors in this model.

## Conclusion

Spatial allocation models are increasingly important in the geographic modeling of the EHFs in LAHCs. This paper has developed and validated a two-stage allocation model to identify the optimal location for new EHFs in an LAHC. Two optimal sites (located in Xiang He Garden and An Jia East Garden) were obtained by applying this two-stage allocation model. All the demand points were covered, which illustrates the applicability of the model as well as the designed solution procedure.

The detailed findings from this research are as follows: a) The rationality of the planning standards in Nanjing has been demonstrated; b) data on the EHFs that have been visualized can help decisionmakers incorporate and process as much spatial information as possible to obtain a set of candidate locations; c) with the two objectives of providing both equitable and efficient access to EHFs for the elderly, this bi-objective model enables the optimal locations of EHFs to be obtained through the greedy algorithm; and d) options for the reasonable expansion of EHFs in an LAHC were identified, which can help the government ensure that its policies and the guidelines related to EHFs are followed. The results show that the two-stage spatial allocation model can be extended to the allocation of other public-service facilities in different countries or regions to assist policymakers in providing adequate healthcare services for the elderly. Notably, when applying the proposed model to other countries, the data used in stage one to narrow the scope of the community may vary according to the context of the specific country. Furthermore, the particular concerns of policymakers should be considered in the model.

Motivated by the present work, several interesting research topics could be worth investigating in the future. First, if more accurate information about the way people obtain healthcare services could be made available, the results would be closer to reality. Future research should consider examining different ways for the elderly to reach EHFs. Second, this study analyzes the population demand for spatial accessibility, the present use of the land and the number of existing facilities. It would be interesting to examine other parameters, such as the social and esteem needs of the elderly for different types of healthcare services, the quality of services provided and the choices of family members, when developing policies for investment in EHFs.

## Abbreviations

EHF: elderly healthcare facility
GA: greedy algorithm
GIS: geographic information system
LAHC: large-scale affordable housing community
